# Probing Melatonin’s Release Pattern from Oral Solid Dosage Forms Using Synthetic Biopolymers

**DOI:** 10.3390/polym18202570

**Published:** 2026-10-19

**Authors:** Angeliki Siamidi, Nikolaos Bikiaris, Chrystalla Protopapa, Maria Plyta, Maria Saltou, Aikaterini Sakellaropoulou, Marilena Vlachou

**Affiliations:** 1 Section of Pharmaceutical Technology, Department of Pharmacy, National and Kapodistrian University of Athens, 15771 Athens, Greece; asiamidi@pharm.uoa.gr (A.S.); cprotopapa@pharm.uoa.gr (C.P.); mariaplyta03@gmail.com (M.P.); mariasaltou1103@gmail.com (M.S.); 2 Laboratory of Polymer Chemistry and Technology, Department of Chemistry, Aristotle University of Thessaloniki, GR-541 24 Thessaloniki, Greece; nbikiaris@gmail.com; 3 Section of Pharmaceutical Chemistry, Department of Pharmacy, National and Kapodistrian University of Athens, 15771 Athens, Greece; aiksakell@pharm.uoa.gr

**Keywords:** melatonin, drug release, polylactic acid, polyethylene glycol, polymers, copolymers

## Abstract

Melatonin (*N*-acetyl-5-methoxytryptamine) (MLT) is a naturally occurring hormone produced endogenously, primarily in the suprachiasmatic nucleus. MLT exhibits a plethora of uses particularly in the treatment of sleep disorders (sleep onset and maintenance). However, due to its short biological half-life, MLT cannot maintain effective plasma concentrations for a prolonged period when it is exogenously taken from immediate-release formulations. The present study aims at developing modified release systems capable of gradually releasing MLT, thereby sustaining its pharmacological effect over eight hours. To this end, modified-release tablets were prepared using new polymeric excipients based on polylactic acid (PLA). Both neat PLA and PLA copolymers with polyethylene glycols (PEGs) of varying molecular weights (PLLA-PEG-PLLA_4000, PLLA-PEG-PLLA_6000, and PLLA-PEG-PLLA_8000) were used, along with other excipients. Certain formulations delivered MLT comparatively fast and may therefore be useful for sleep onset problems, others matched the release behaviour of the marketed product Circadin^®^, and the rest released the hormone in a more prolonged manner.

## 1. Introduction

Sleep disturbances represent an important public health challenge, with recent epidemiological data estimating that 16.2% of the global adult population meets the criteria for insomnia disorder [1]. Although conventional sedative and hypnotic agents, including benzodiazepines, are widely used in the management of insomnia, their long-term use is restricted by adverse effects, such as tolerance, dependence, residual daytime sedation, and cognitive impairment [2]. These limitations have increased the interest towards alternative approaches for managing insomnia that may offer a more favourable safety profile while supporting physiological sleep mechanisms [3].

In this context, melatonin has attracted increasing interest as an alternative approach for the management of sleep disorders, with its use having increased considerably over recent decades [4]. As an endogenous hormone with an important role in the regulation of circadian rhythms and sleep timing, exogenous MLT can contribute to the synchronization of the sleep–wake cycle [5]. However, the oral administration of MLT is limited by its low aqueous solubility, extensive first-pass metabolism, short systemic half-life (45 min), and low and variable oral bioavailability (9–33%, with a mean value of approximately 15%) [6,7]. These limitations have stimulated the development of advanced drug delivery systems designed to improve MLT bioavailability and provide controlled release profiles that more closely mimic its physiological nocturnal secretion [8].

Over the past fifteen years, our research group has been involved in formulating new oral MLT solid dosage forms with different MLT release characteristics that target both sleep onset and sleep maintenance dysfunctions [9,10,11,12]. Attention has been given to modified-release MLT formulations, which may be advantageous when prolonged MLT exposure is required to support sleep maintenance [13]. Furthermore, the low and variable oral bioavailability and short systemic half-life of MLT provide a rationale for the development of sustained-release formulations capable of maintaining therapeutically relevant concentrations over an extended period [7,8].

Biodegradable aliphatic polyesters, and poly(lactic acid) (PLA) in particular, are attractive excipients for such systems, owing to their biocompatibility, renewable origin, and regulatory acceptance and the wide tunability of their thermal and physicochemical properties through copolymerization [14,15]. In our previous studies, PLA and its copolymers with “softer” aliphatic polyesters, such as poly(ethylene adipate), poly(butylene adipate) and poly(ε-caprolactone), proved to be effective co-formulants in MLT matrix tablets, with the composition of the copolymer modulating both the extent and the rate of MLT release [9,11,16]. Block copolymers of PLA with poly(ethylene glycol) (PEG) constitute another extensively investigated family, since the combination of a hydrophobic, crystallizable polyester with a hydrophilic, non-toxic polyether yields amphiphilic macromolecules capable of self-assembling into micelles, nanoparticles and polymersomes [17,18,19]. Triblock copolymers of PLA and PEG, in particular, have been exploited since the pioneering work of Jeong et al. on injectable thermoreversible gels [20,21], mainly in the form of thermoresponsive or stereocomplexed hydrogels [22,23,24], including, in a recent study by some of us, for the sustained delivery of the iron chelator deferoxamine [25]. In practically all of these systems, however, the copolymers are of relatively low molecular weight and rich in PEG, so their function relies on self-organization in aqueous media rather than on the properties of the bulk polymer.

A distinctly different class of PLA-*b*-PEG-*b*-PLA triblocks has recently been introduced in previous works [26,27]. In the first series with fixed PEG blocks and lactide/PEG molar ratios from 20/1 to 640/1, PEG was shown to act as a plasticizer of PLA, lowering its glass transition temperature (Tg) and increasing the free volume with increasing PEG content [26]. More recently, keeping the lactide/PEG ratio fixed at 640/1 (i.e., one PEG chain per 640 lactide molecules, corresponding to only a few wt% of PEG) and varying the molecular weight of PEG from 1000 to 8000 g/mol, we demonstrated that the length of the PEG block is the critical parameter: whereas PEG1000 did not affect PLA, PEG blocks of 4000–8000 g/mol systematically lowered *T*g by more than 15 K (down to 35 °C) [27]. From a drug delivery standpoint, the novelty of these triblocks lies in the fact that PEG functions as a covalently bound, non-migrating internal plasticizer, contrary to conventional low-molecular-weight plasticizers of PLA, which are prone to migration and leaching [28,29], bringing the *T*g of an essentially PLA-based material close to, or even below, body temperature, while the high molecular weight required for mechanical integrity and processability is retained. Since chain mobility, free volume, crystallinity and semicrystalline morphology are the very parameters that govern water uptake, drug diffusivity and hydrolytic degradation in polyester matrices [30,31], these copolymers offer a molecular handle for modulating the release rate without altering the chemical composition of the carrier.

On these grounds, we hypothesized that the systematic dependence of the thermal properties and molecular dynamics of these triblocks on the length of the PEG block could be translated into the corresponding, and therefore tunable, dependence of the MLT release kinetics. Accordingly, the three copolymers in which the plasticizing effect of PEG was clearly manifested, namely PLA-*b*-PEG(4000)-*b*-PLA, PLA-*b*-PEG(6000)-*b*-PLA and PLA-*b*-PEG(8000)-*b*-PLA, were selected and employed herein, for the first time, as matrix tablets for MLT, with the specific objective of evaluating the effect of the molecular weight of the PEG block on the release behaviour of the hormone. MLT has previously been shown to be well compatible with PLA-based matrices [16], whereas the hydrophilic PEG segments, together with the lower *T*g, were expected to facilitate water penetration and drug diffusion, thus offering a rationale for tailoring the release of MLT over the desired time window. To the best of our knowledge, PLA-rich triblocks of this type have not previously been investigated as release-controlling excipients for MLT. The present study therefore aimed at developing formulations in which these copolymers deliver the hormone gradually over an eight-hour period so as to potentially support both sleep onset and sleep maintenance. To this end, the influence of the formulation composition on the MLT release profile was investigated, in order to identify the best systems that meet this target.

## 2. Materials and Methods

Melatonin (MLT) was purchased from Tokyo Chemical Industry (Tokyo, Japan). Poly(ethylene glycol) (PEG) was used as the starting block for the synthesis of the triblock copolymers. PEG samples of varying molecular weights (4000, 6000 and 8000 g/mol) were purchased from Aldrich Co. (London, UK). A stannous octoate (Sn(Oct)_2_) catalyst was also obtained from the same supplier. High-purity *L*-lactide (99.9%) was sourced from PURAC Biochem BV (Gorinchem, The Netherlands) under the trade name PURASORB^®^L. Hydroxypropylmethylcellulose (HPMC K15M) was obtained from Sigma-Aldrich (Steinheim, Germany), alginic acid sodium salt (low viscosity) and microcrystalline cellulose (MCC) (Avicel PH 102) from Alfa Aesar GmbH & Co. KG (Karlsruhe, Germany), lactose monohydrate from Merck (Darmstadt, Germany), and magnesium stearate from Riedel-De Haen (Hannover, Germany). The commercially available drug Circadin^®^ (RAD Neurim Pharmaceuticals EEC SARL) was purchased from a local pharmacy store. All chemicals used in this study were of reagent grade and did not require any additional purification.

### 2.1. Synthesis of PLA and PLLA_PEG_PLLA Triblock Copolymers

The ring-opening polymerization (ROP) of L-lactide yielded ABA triblock PLLA-PEG-PLLA copolymers, utilizing three distinct PEG molecular weights, namely 4000, 6000, and 8000 g/mol. PEG and L-lactide in a molar ratio of 640/ 1 were fed into a glass batch reactor along with 400 ppm of a Sn(Oct_2_) (as to *L*-lactide) catalyst dissolved in toluene at a concentration of 20 mg/mL. The reactor was repeatedly evacuated and filled with N_2_ at least three times to remove air, as completely as possible. The copolymerization mixture was subjected to heating at 160 °C for 3 h under a nitrogen flow of 5 mm^3^ /min, with the stirring rotation speed fixed at 200 rpm. The process was followed by an additional 1 h of heating at 180 °C. In the final step, the reaction chamber was gradually evacuated for approximately 15 min to remove unreacted lactide monomers. Polymerization was terminated by rapidly cooling the flask to ambient temperature, and white bulk solid products were obtained in all cases. To provide a control, neat PLA was produced using the same ROP method, temperature, and duration. Therefore, a total of four (4) polymers were successfully synthesized.

### 2.2. Recrystallization of PLA and Copolymers

The requisite polymer, PLA, PLLA-PEG-PLLA_4000, PLLA-PEG-PLLA_6000 and PLLA-PEG-PLLA_8000 (1.45 g), in the form of large hard chunks, were sonicated with a mixture of ethyl acetate (25 mL) and dichloromethane (15 mL) at 35 °C for 30 min. The resulting clear solution was then treated dropwise with n-pentane at ambient temperature, until an off-white solid was precipitated. The solid was filtered in vacuo, washed with n-pentane (2 × 10 mL), and dried under vacuum in open air to quantitatively give the respective polymer, as a white powder, which was used in this form for the preparation of the matrix tablets.

### 2.3. Gel Permeation Chromatography (GPC)

The molecular weights of the samples were assessed via Gel Permeation Chromatography/Size Exclusion Chromatography (GPC/SEC). Analysis was carried out using an Agilent 1260 Infinity II LC system (Agilent Technologies, Santa Clara, CA, USA) equipped with an isocratic G7110B pump, a G7129A autosampler, a G7162A Refractive Index Detector (RID), and a PLgel 5 µm (50 × 7.5 mm) guard column in series with two PLgel 5 µm (300 × 7.5 mm) MIXED-C columns. For system calibration, three poly(methyl methacrylate) (PMMA) standards with molecular weights ranging from 0.535 to 1591 kg/mol were utilized. Sample solutions were prepared at a concentration of 3 mg/mL and filtered through PTFE membranes with a pore size of 0.45 µm. Each run involved an injection volume of 20 μL and a total analysis time of 30 min. Both the columns and the RID were maintained at a constant temperature of 40 °C.

### 2.4. Nuclear Magnetic Resonance (^1^H NMR)

The NMR spectra of the prepared copolymers were recorded using an Agilent spectrometer (Santa Clara, CA, USA) operating at 500 MHz for proton analysis under room temperature conditions. For sample preparation, deuterated chloroform (CDCl_3_) was used as the solvent, forming 5% *w*/*v* solutions. Acquisition involved 32 scans for ^1^H NMR and 512 scans for ^13^C NMR. Additionally, a uniform sweep width of 6 kHz was consistently applied in all measurements.

### 2.5. Attenuated Total Reflection Fourier Transform Infrared (ATR-FTIR) Spectroscopy

ATR-FTIR spectra for all samples were acquired using a Shimadzu IRTracer-100 spectrophotometer (Kyoto, Japan) equipped with a QATR™ 10 Single-Reflection (Kyoto, Japan) ATR accessory featuring a diamond crystal. Measurements were conducted in absorbance mode within the spectral range of 450–4000 cm^−1^ at a resolution of 2 cm^−1^. Each spectrum was produced by averaging 32 scans. Prior to analysis, the spectra underwent baseline correction and normalization.

### 2.6. Differential Scanning Calorimetry (DSC)

The glass transition, crystallization, and melting events were assessed by conventional calorimetry using a TA Q200 DSC apparatus (TA Instruments, New Castle, DE, USA). The instrument was initially cleaned well and calibrated with sapphires for heat capacity and indium for temperature and enthalpy. Samples of ∼7–9 mg in mass were closed in aluminum TZero TA pans. Measurements were carried out in a high-purity nitrogen atmosphere and in the temperature range from −100 to 190 °C. An initial heating scan from room temperature to 190 °C was performed to erase the thermal history and ensure good thermal contact between the sample and the pan. The samples were then rapidly cooled at non-constant rates of 60–100 K/min, using the “jump” command in TA software (Version 5.1), and heated at 10 K/min (scan 1), in order to record the glass transition and cold crystallization of the initially amorphous samples. Subsequently, the samples were cooled at 20 K/min and heated again at 10 K/min (scan 2), in order to record their melt crystallization and the thermal transitions of the semicrystalline materials. T_g_ (half heat capacity change, Δc_p_/2, method) and T_m_ were determined from the heating scans, while the crystallization temperature, T_c_, and enthalpy, ΔH_c_, were determined from the cooling scan at 20 K/min.

### 2.7. X-Ray Diffraction (XRD)

X-ray diffraction (XRD) measurements were conducted at room temperature across a 2θ range of 5° to 50°, using a scanning rate of 1°/min. The analysis was performed with a MiniFlex II diffractometer (Chalgrove, Oxford, UK), employing Cu Ka radiation (λ = 0.154 nm) for crystalline phase identification.

### 2.8. Enzymatic Degradation

For enzymatic degradation testing, thin films prepared by compression moulding (thickness 10–15 mm) were cut in squares with dimensions ~1 cm × 1 cm and were placed in test tubes containing 10 mL phosphate-buffered solution (pH 7.4) with 0.01 mg/mL lipase from Pseudomonas cepacia and 0.1 mg/mL lipase from Rhizopus oryzae. This combination of lipases was chosen because it is commonly employed to evaluate the enzymatic degradation of aliphatic polyesters such as PLA and its copolymers so that the present results can be compared directly with earlier studies performed under the same conditions. Moreover, lipases are ester-cleaving enzymes found in the gastrointestinal tract and are therefore pertinent to orally administered dosage forms. Flasks were then incubated at 37.1 ± 1 °C in an oven for several days, while media were replaced every 3 days. After a specific period of incubation (days 7, 13, 19 and 26), the samples were removed from the flasks, washed with distilled water, dried under vacuum at 30 °C for 24 h, and weighed until they remained at a constant weight. The degree of enzymatic hydrolysis was estimated from the mass loss of the samples. Blank incubations were also carried out with the samples in the same buffer without enzyme addition. The degree of gravimetric loss was calculated by the sample weight loss, according to the following equation:

(1) $$Mass\;loss\;\%=\frac{W_{0}\;-{\;W}_{i}}{W_{o}}$$

Each weight loss was measured in triplicate, and the mean value was calculated.

### 2.9. Tablet Production

Tablets were prepared by accurately weighing MLT and the excipients, as shown in Table 1, using an ATX224^®^ high-precision analytical balance (Shimadzu, Kyoto, Japan). The weighed ingredients were subsequently blended in a laboratory-scale powder blender (32 rpm, 8 min) in a Turbula^®^ T2F blender mixer (WAB, Nidderau, Germany). Magnesium stearate, which was used as a lubricant, was then added, and mixing continued for a further 2 min. The produced mixture was precisely weighed (200 mg), filled to a 10 mm diameter dye, and directly compressed using an MP 150 hydraulic press (Maasen GmbH, Möglingen, Germany).

### 2.10. Tablet Weight Variation and Physical Characterization

Tablet uniformity tests were performed on all new formulations (F1–F6). Mass uniformity was assessed using an ATX224^®^ analytical balance (Shimadzu, Kyoto, Japan).

Tablet thickness was determined utilizing a Vernier calliper scale.

Tablet hardness was assessed using a THB 28 hardness tester (ERWEKA GmbH, Langen, Germany). The force generated (measured in N) was sufficient to break the tablet [32].

Tablet friability was assessed using a type TA 3R test apparatus (Erweka, Heusenstamm, Germany). Ten tablets were cleaned to remove any excess dust, accurately weighed, and set in the friability spinning drum. The drum was turned for 4 min at a speed of 25 rpm. The tablets were de-dusted and weighed once again. The friability percentage was expressed using the corresponding Equation (2):

Friability = [(Initial weight − Final weight)/Initial weight] × 100%(2)

Uncoated compressed tablets that exhibited a weight loss of less than 1% after 100 revolutions were regarded as acceptable [32].

### 2.11. Dissolution Test

Dissolution tests were performed on all formulations produced (F1–F6) and the commercially available drug Circadin^®^, for comparison purposes. The tests were conducted using a PT-DT7 USP Type II dissolution apparatus (Pharma Test, Hainburg, Germany) operating at 37 ± 0.5 °C and 50 rpm. To mimic the fed stomach environment, an aqueous medium at pH 4.5 was utilized. At certain time points, 5 mL aliquots were collected, filtered through Titan3™ 0.45 μm regenerated cellulose syringe filters (Thermo Fisher Scientific Inc., Rockwood, TN, USA), and analyzed at a wavelength of 278 nm using a uniSPEC 2 UV–Vis spectrophotometer (LLG Labware, Meckenheim, Germany).

### 2.12. Equation Fitting

To characterize the release kinetics, the in vitro dissolution data were fitted to four commonly applied mathematical models [33]:

Zero-order equation:

C = k_0_·t (3)

in which k_0_ denotes the zero-order release constant and t the release time.

First-order equation:

log C = log C_0_ − k·t/2.303 (4)

in which C_0_ represents the initial drug concentration and k the first-order rate constant.

Higuchi square-root equation:

Q = K·t^0.5^ (5)

with K being a constant that accounts for the design parameters of the system.

Korsmeyer–Peppas equation:

*M_t_*/*M_∞_* = *kt^n^* (6)

where M_t_ and M_∞_ represent the cumulative amount of drug released at time t and at infinite time, respectively. The parameter k denotes the release rate constant, while *n* is the diffusion exponent. The value of n provides insights into the drug release mechanism from tablets under perfect sink conditions. For cylindrical tablets, such as those investigated in the present study, a value of n = 0.45 indicates a Fickian diffusion release mechanism (case I diffusional), 0.45 < n < 0.89 signifies anomalous non-Fickian transport, and when n = 0.89, it represents zero-order (case II) release kinetics.

The Korsmeyer–Peppas model was specifically chosen for this study as it effectively characterizes drug release behaviour from polymeric systems, particularly when multiple mechanisms of release are involved.

### 2.13. Statistical Analysis

Gravimetric weight loss values were analyzed using a repeated-measures ANOVA, applying the Greenhouse–Geisser correction to adjust for violations of the sphericity assumption. Post hoc pairwise comparisons were adjusted using the Bonferroni correction, while Tukey’s correction was used for multi-group comparisons. All data are presented as the mean ± SD, and *p* < 0.05 was considered statistically significant. All analyses were performed using IBM SPSS Statistics v28.0.

## 3. Results

### 3.1. Structural Investigation of PLA Polymers

Based on GPC/SEC analysis, the number-average molecular weights (M_n_) of the PLLA–PEG–PLLA copolymers were estimated to be 108, 62, and 101 kg/mol for samples prepared using PEG segments of 4000, 6000, and 8000 g/mol, respectively (Table 2). Under otherwise comparable ring-opening polymerization (ROP) conditions, the polymerization of *L*-lactide in the absence of PEG yielded neat PLA with an M_n_ of approximately 82 kg/mol.

**Figure 1:**
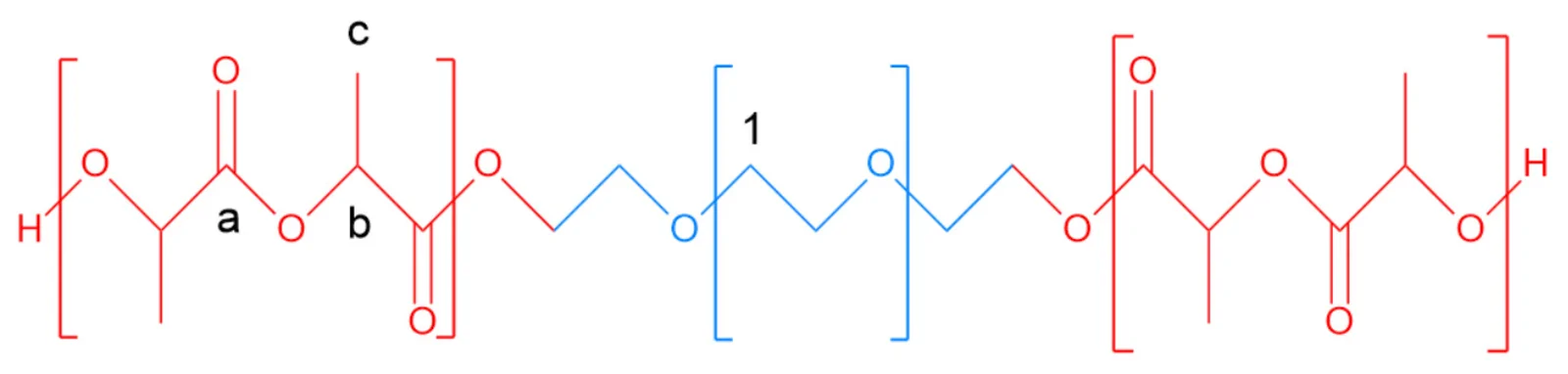
A schematic representation of the ABA-type PLLA–PEG–PLLA triblock copolymer architecture, consisting of hydrophobic PLA terminal blocks (red) and a hydrophilic PEG central block (blue).

**Figure 2:**
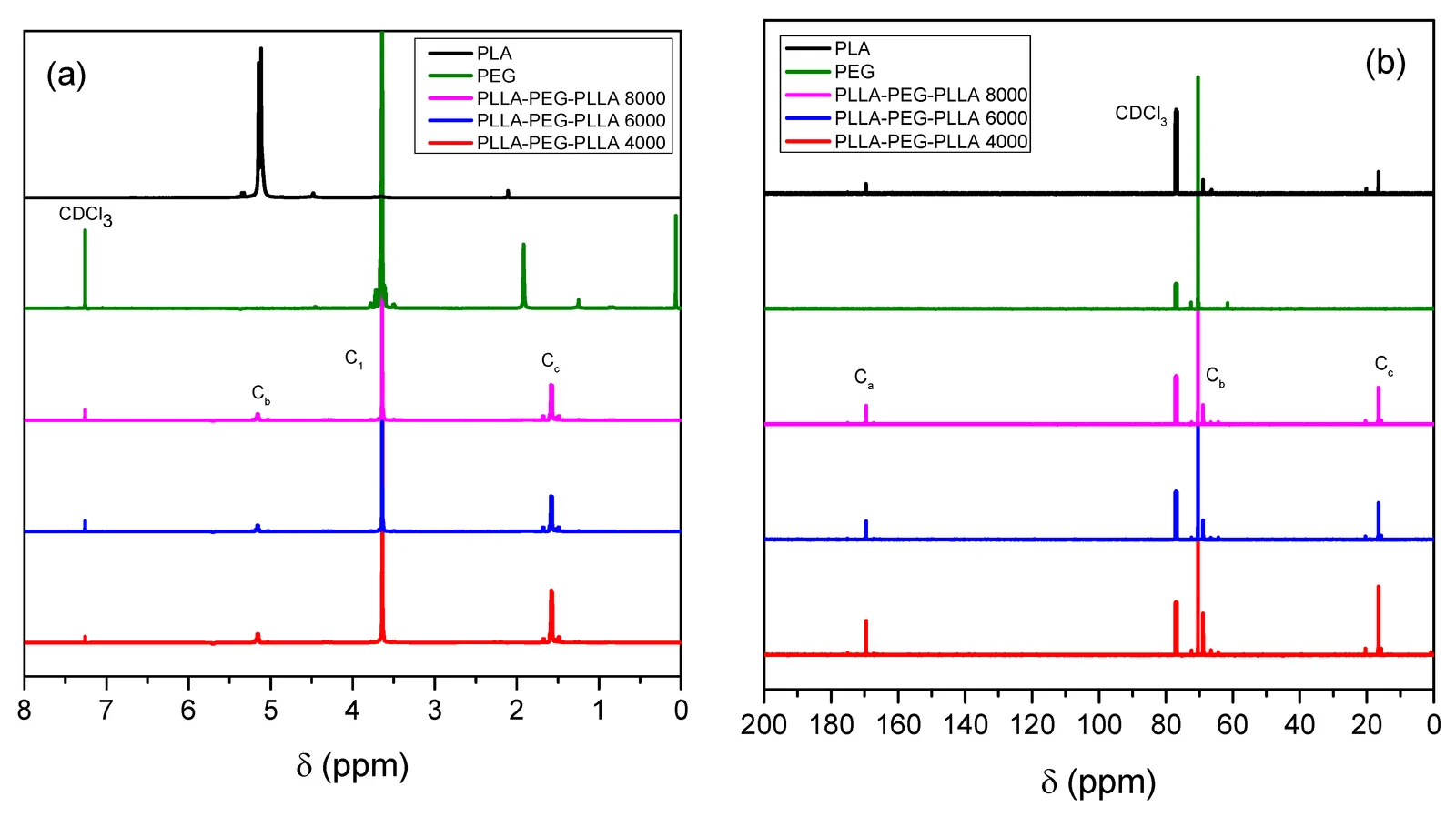
(**a**) ^1^H NMR and (**b**) ^13^C NMR spectra of pure PLA, PEG and PLLA_PEG_PLLA_4000, PLLA_PEG_PLLA_6000, and PLLA_PEG_PLLA_8000 triblock copolymers.

NMR was employed herein to confirm the successful synthesis of the PLLA-PEG-PLLA copolymers, providing structural information on the PLA and PEG segments by assigning chemical shifts to specific protons and carbons. ^1^H NMR and ^13^C NMR spectroscopy were initially performed on both PLA and PEG to identify their characteristic peaks. The expected ABA-type structure is depicted in Figure 1. The NMR spectra confirm the characteristic chemical structures of pristine PLA and the PLLA–PEG–PLLA triblock copolymers. For neat PLA, the ^1^HNMR spectrum (Figure 2a) exhibits the expected resonances at 5.2 and 1.8 ppm, assigned to the methine ($H_{b}$) and methyl ($H_{c}$) protons, respectively. In the spectra of the PLLA-PEG-PLLA triblock copolymers, the strong signal at 3.6 ppm is due to the PEG methylene protons (H_1_) of the –CH_2_CH_2_O– repeat unit, whereas the PLA blocks give rise to the signals at 5.1 ppm (methine proton neighbouring the ester oxygen, C_b_) and 1.5 ppm (methyl protons, C_c_). The ^13^C NMR spectrum (Figure 2b) presents three main signals at approximately 170, 68, and 18 ppm, corresponding to the ester carbonyl carbon ($C_{a}$), the oxygen-bearing methine carbon ($C_{b}$, CH–O), and the methyl carbon ($C_{c}$, CH_3_), respectively.

**Figure 3:**
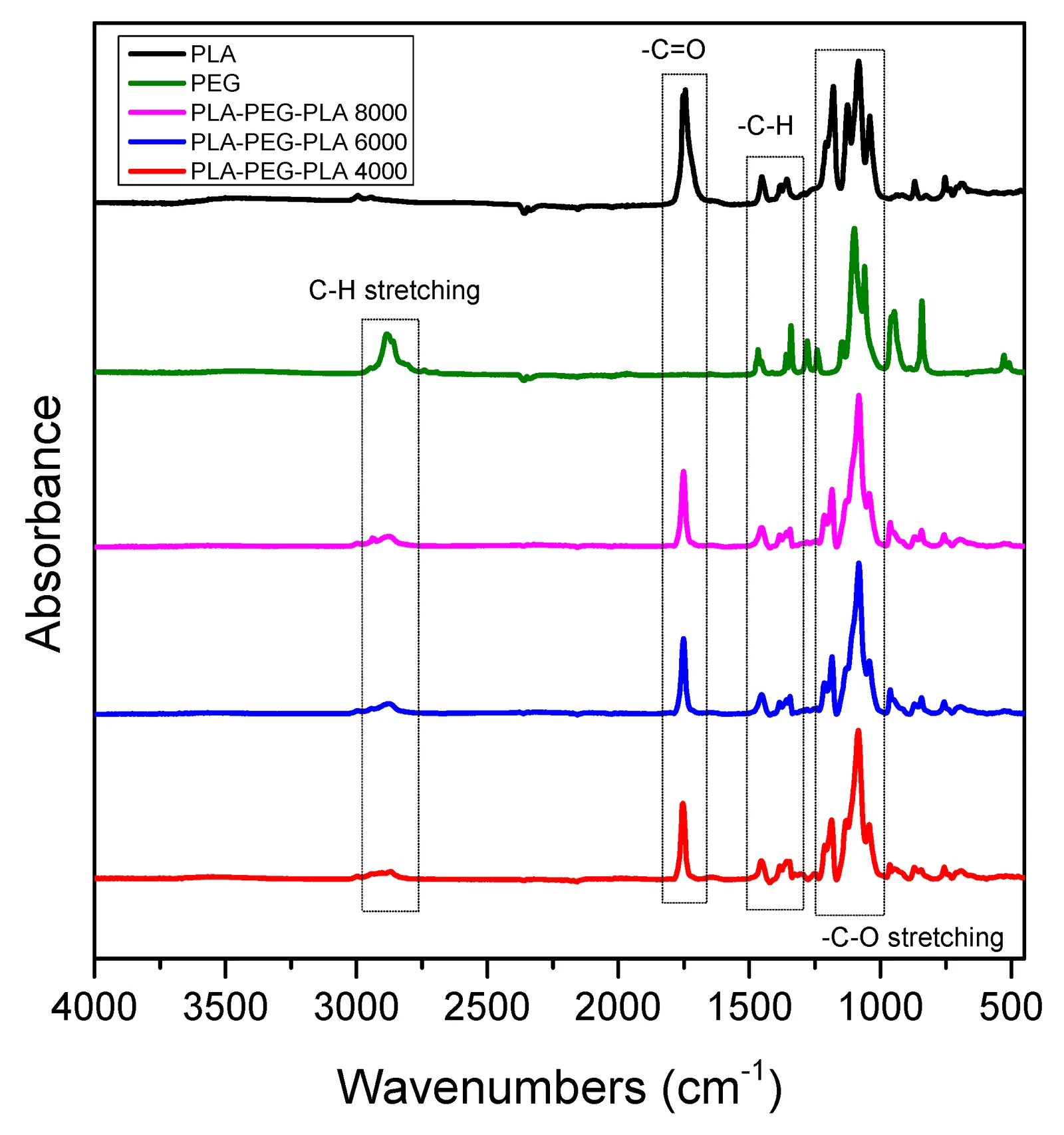
ATR–FTIR spectra of pure PLA, PEG and PLLA_PEG_PLLA_4000, PLLA_PEG_PLLA_6000, and PLLA_PEG_PLLA_8000 triblock copolymers.

ATR-FTIR spectroscopy (Figure 3) was additionally employed to verify that PLA had been formed via ROP and to examine the structure of the copolymers. Owing to the high LA/PEG ratio (640/1), the spectra of the copolymers are very similar to that of neat PLA. The absorption bands arising from the vibrations of the different bonds (C–H, C–O and C=O) are marked in Figure 3 and agree with those reported for PLA in the literature. Based on these data, no additional bands attributable to the triblock structure could be distinguished. The spectrum of neat PLA displays absorption bands at approximately 3000–2850 cm^−1^ (C–H stretching), a sharp peak at 1744 cm^−1^ (C=O stretching of the ester groups), bands at 1455 cm^−1^ (bending vibrations of the –CH_2_ and –CH_3_ groups) and a strong band around 1080 cm^−1^ (C–O stretching of the ester bond) [19]. The spectra of the PLLA-PEG-PLLA copolymers are dominated by the same PLA-related bands, as expected from the high LA/PEG ratio, whereas the contribution of the ether (C–O–C) bonds of the PEG block is manifested as additional absorption in the 1200–1000 cm^−1^ region [34,35]. No new absorption bands were detected, confirming that copolymerization proceeded without side reactions.

### 3.2. Investigation of Thermal Transitions (DSC)

**Figure 4:**
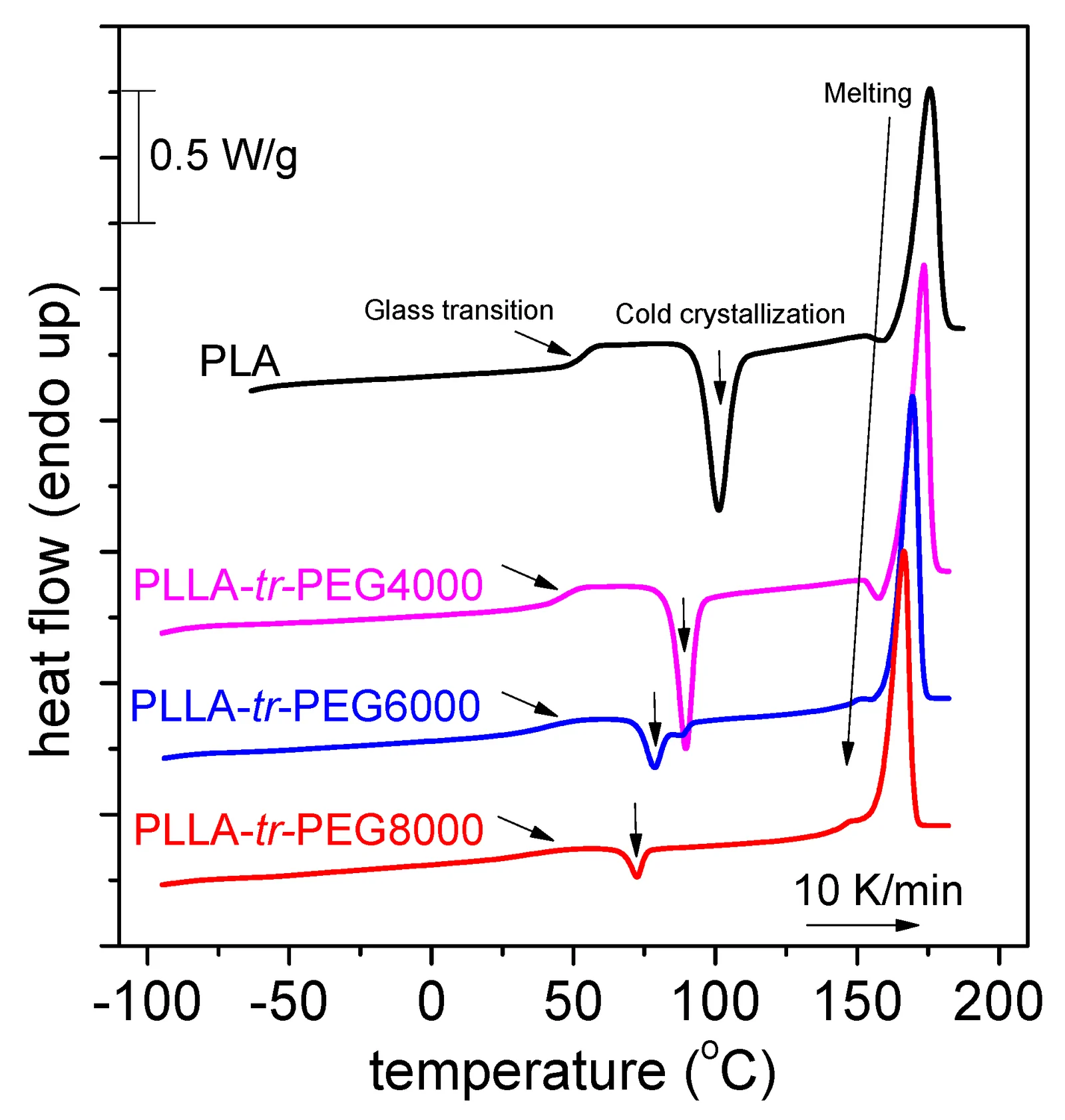
Comparative DSC heating scans (10 K/min) of the initially amorphous (rapidly cooled) samples. The arrows indicate the effects recorded during the various thermal events (glass transition, cold crystallization, melting). The heat flow is presented upon normalization to the mass of each sample.

The thermal transitions of neat PLA and the PLLA-PEG-PLLA triblock copolymers were evaluated by DSC. The heating scans of the initially amorphous (rapidly cooled) samples are shown in Figure 4, while the values determined for the semicrystalline samples (scan 2) are summarized in Table 3. Single and clear glass transition steps are recorded for all samples (Figure 4). The characteristic temperature, *T*_g_, decreases from 53 °C in neat PLA to 46 °C in PLLA-PEG-PLLA_4000 and further to 39 and 35 °C in PLLA-PEG-PLLA_6000 and PLLA-PEG-PLLA_8000, respectively (Table 3); practically the same values (52, 46, 40 and 35 °C) were obtained for the amorphous samples. Thus, *T*_g_ drops systematically, by up to 18 K, with increasing MW_PEG_, despite the very low PEG content of the copolymers, which reflects the plasticizing action of the PEG block on the PLA chains. Consistently with the enhanced chain mobility, the cold crystallization of PLA upon heating (Figure 4) shifts to progressively lower temperatures in the copolymers, and upon cooling from the melt at 20 K/min, the copolymers crystallize at slightly lower temperatures than neat PLA (*T*_c_ = 91–95 °C vs. 96 °C) but with a markedly higher enthalpy, Δ*H*_c_, increasing from 2 J/g in neat PLA to 8, 24 and 30 J/g in the PEG 4000-, 6000- and 8000-based copolymers, respectively (Table 3) [36]. Finally, the melting temperature, *T*_m_, decreases moderately from 176 °C in neat PLA to 174, 169 and 166 °C with increasing MW_PEG_, suggesting the formation of smaller and/or less perfect crystals in the copolymers, in agreement with our previous detailed study on these materials [27].

### 3.3. Crystalline Structure Assessment

**Figure 5:**
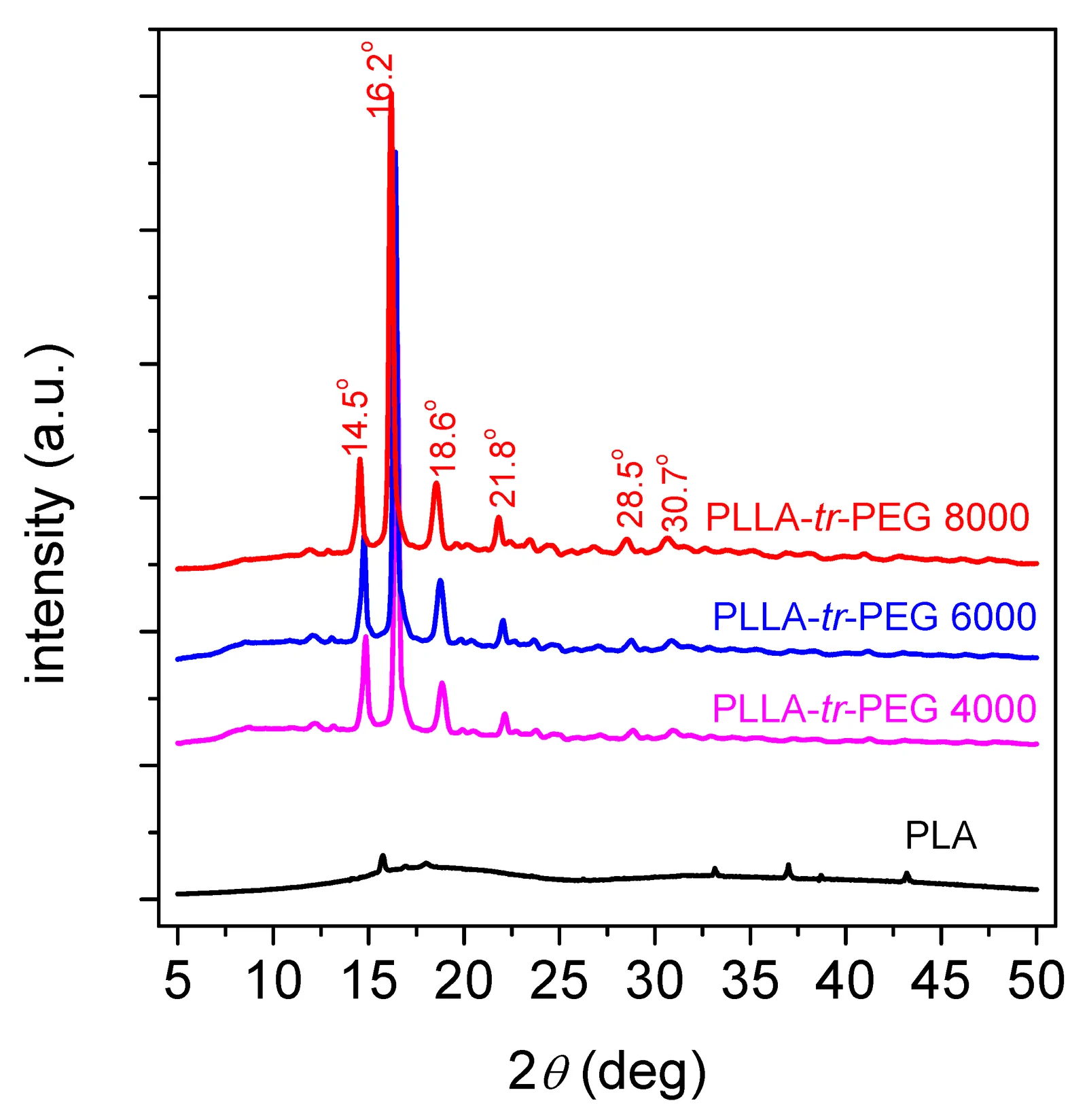
X-ray diffraction spectra of PLLA-PEG-PLLA triblock copolymers with different PEG segments.

The XRD patterns of neat PLA and the copolymers in their as-synthesized state are shown in Figure 5. Neat PLA exhibits only a broad amorphous halo with very weak reflections, i.e., it was recovered from the reactor in a practically amorphous state, consistent with the slow crystallization of high-molecular-weight PLA. In contrast, all three PLLA-PEG-PLLA copolymers display the characteristic reflections of crystalline PLLA at 2θ ≈ 14.5°, 16.2°, 18.6° and 21.8°, together with weaker reflections at 28.5° and 30.7° [34,35,36], the intensity of which increases in the order PLLA-PEG-PLLA_4000 < PLLA-PEG-PLLA_6000 < PLLA-PEG-PLLA_8000. No reflections attributable to crystalline PEG (expected at 2θ ≈ 19° and 23°) [34,35] are detected, in line with the very low PEG content and the suppression of PEG crystallization within the triblock architecture [25,27].

### 3.4. Hydrolysis Under Enzymatic Conditions

**Figure 6:**
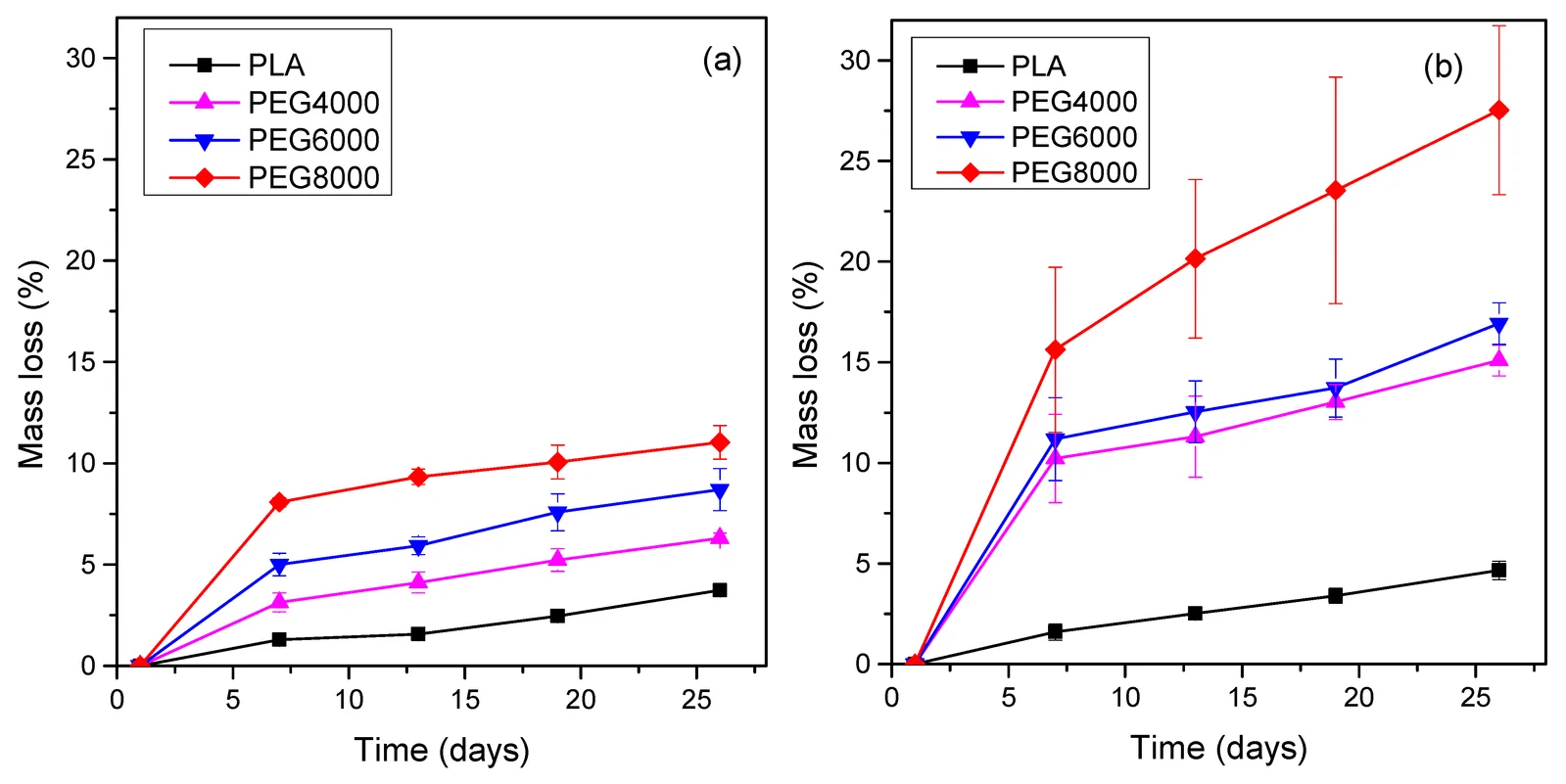
The mass loss (%) of neat PLA and the PLLA-PEG-PLLA triblock copolymers during 26 days of incubation at 37 °C in PBS (**a**) without and (**b**) with enzymes. Data are the means of three independent experiments; error bars represent standard deviations.

Cumulative mass-loss profiles under hydrolytic and enzymatic conditions over 26 days are shown in Figure 6. A one-way ANOVA revealed a significant effect of composition at every sampling point under both conditions (*p* ≤ 0.002), and a repeated-measures ANOVA confirmed the significant effects of both composition and time in both media (*p* < 0.001), with a significant composition × time interaction only under enzymatic conditions (*p* = 0.019, Greenhouse–Geisser-corrected). Under hydrolytic conditions (Figure 6a), neat PLA was the most resistant sample, losing only 3.74 ± 0.32% of its mass after 26 days, owing to its hydrophobic character and the slow hydrolysis of its ester bonds [37], whereas the mass loss of the copolymers increased with the molecular weight of PEG, reaching 6.30 ± 0.28%, 8.70 ± 1.40% and 11.03 ± 0.83% for PLLA-PEG-PLLA_4000, _6000 and _8000, respectively; on day 26, all samples differed significantly from one another. In the presence of the enzymes (Figure 6b), the degradation kinetics changed fundamentally. After 26 days, neat PLA exhibited only 4.66 ± 0.46% mass loss, whereas PLLA-PEG-PLLA_4000, _6000 and _8000 exhibited 15.09 ± 0.76%, 16.93 ± 1.03% and 27.53 ± 7.50%, respectively. PLLA-PEG-PLLA_8000 showed significantly higher mass loss than all other samples from day 19 onwards, and on day 26, the mass loss of PLLA-PEG-PLLA_4000 and _6000 also significantly exceeded that of neat PLA.

### 3.5. Results of Tablet Weight Variation Tests and Physical Characterization of Tablets

The results of the tablet uniformity test were considered acceptable. Specifically, the following were found:

The uniformity of mass revealed a mean weight of 200 ± 2.4 mg.

The thickness test revealed that the tablet diameter was 10 mm, and tablet thickness was 2.6 ± 0.8 mm for all formulations.

The hardness test showed that tablet surface hardness was found to be between 86 and 98 N.

The friability test was regarded as acceptable as the test findings demonstrated that tablet friability was consistently less than 1%, indicating sufficient tablet strength.

### 3.6. Dissolution Test Results and Equation Fitting

**Figure 7:**
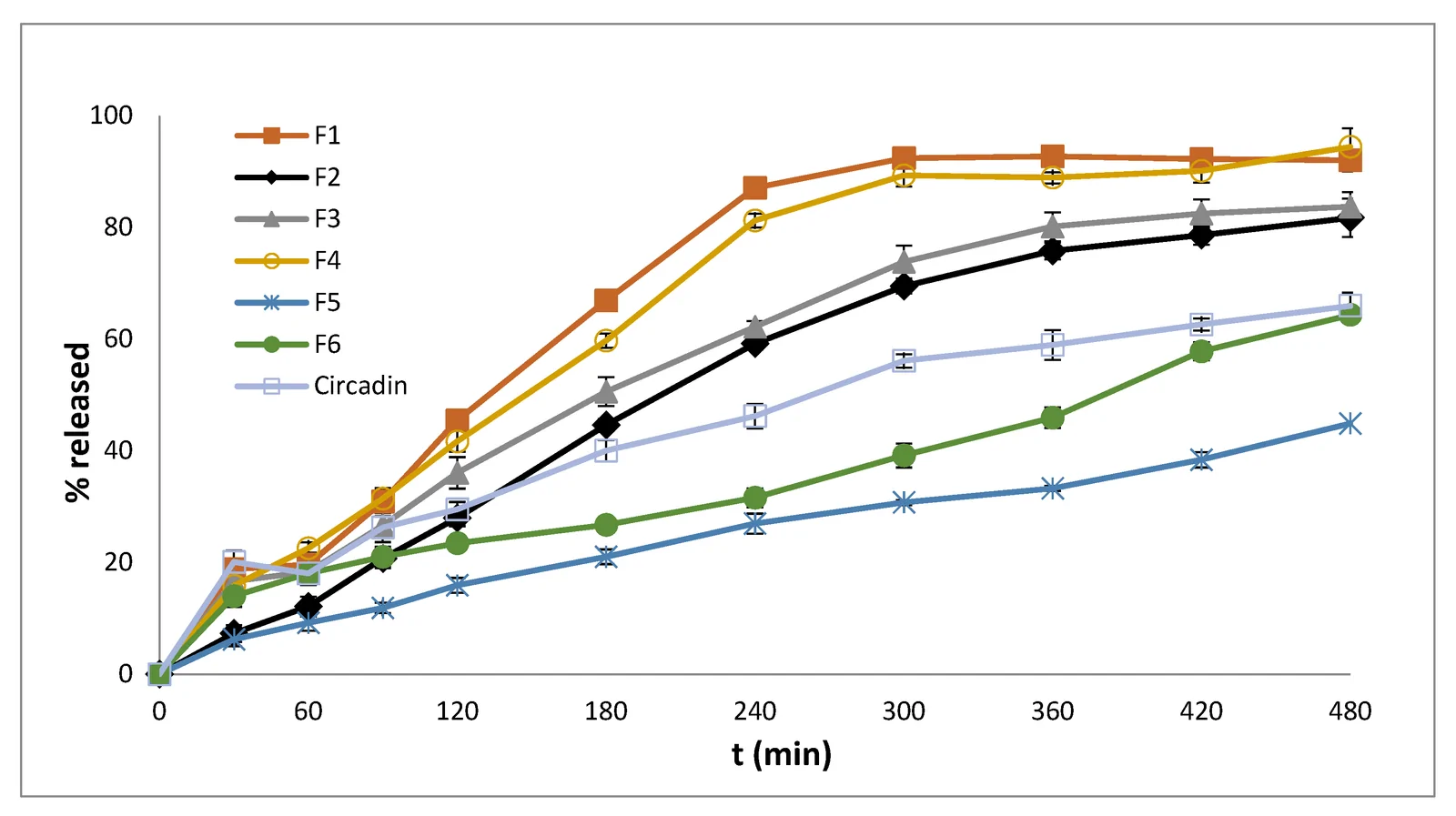
The cumulative release (%) of MLT (mean ± SD, n = 3) as a function of time from the prepared formulations and the marketed product Circadin^®^.

The following Figure 7 demonstrates the in vitro % release of MLT from the formulations F1–F6 and the commercially available drug Circadin^®^ vs. time.

The outcomes of the kinetic model fitting are summarized in Table 4. The Korsmeyer–Peppas model fitted the release data of all formulations, as well as those of Circadin^®^, satisfactorily (R^2^ = 0.95–0.99). In addition, the zero-order model provided a good fit for F5, F6 and Circadin^®^ (R^2^ = 0.97–0.99), implying a nearly constant release rate throughout the tested period.

## 4. Discussion

The terminal hydroxyl groups (-OH) of the PEG macroinitiator enabled the growth of two PLA chains, one from each end of the PEG block, thereby affording PLLA–PEG–PLLA triblock copolymers. A series of three copolymers was successfully synthesized at a fixed initial LA/PEG molar ratio of 640/1, using PEG precursors with molecular weights between 4 and 8 kg/mol. An increase in the PEG chain length is anticipated to enhance the hydrophilicity and the chain mobility of the copolymer, whereas the long PLA blocks govern its hydrophobic character and mechanical integrity, as well as the extent of drug–polymer interactions.

The successful formation of the PLLA–PEG–PLLA triblock copolymers through the ROP of lactide was initially confirmed by FTIR and NMR spectroscopy (Figure 2 and Figure 3). The spectra of all copolymers were highly similar to that of neat PLA, indicating that the chemical structure of the materials was dominated by the PLLA segments. This finding is consistent with the high initial LA/PEG molar ratio of 640/1, which resulted in a comparatively low PEG content relative to the PLA blocks. Nevertheless, the characteristic PEG-related contributions observed in the copolymer spectra confirm the incorporation of the PEG macroinitiator into the final polymer architecture. Overall, these results support the successful growth of PLA chains from both hydroxyl-terminated ends of PEG and the formation of the targeted PLLA–PEG–PLLA triblock copolymers.

Apart from PLLA_PEG_PLLA_6000, the copolymers exhibited higher M_n_ values than neat PLA, supporting the formation of PLA chains from both hydroxyl termini of the PEG macroinitiator. However, the molecular weights were not twice that of neat PLA. This difference can be attributed to the different polymerization mechanisms: whereas PLA formation proceeds through initiation at randomly distributed initiator sites, copolymerization is initiated specifically from the PEG end groups. Consequently, two PLA blocks grow simultaneously from the PEG segment, resulting in increased—but not double—molecular weights relative to neat PLA. The lower M_n_ observed for the PEG 6000-based copolymer may indicate that PEG 6000 additionally acted as a coinitiator during ROP. Initiation from a larger number of PEG molecules would generate more PLLA–PEG–PLLA chains, each containing shorter PLA blocks, thereby decreasing the overall M_n_.

The length of the PEG block also markedly affected the crystallization behaviour of the copolymers. Whereas neat PLA was recovered from the reactor in a practically amorphous state, all copolymers were semicrystalline, and the intensity of the PLLA reflections increased with the molecular weight of PEG (Figure 5); consistently, the melt-crystallization enthalpy increased from 2 J/g for neat PLA to 30 J/g for PLLA-PEG-PLLA_8000 (Table 3). Thus, rather than hindering the packing of the PLA segments, the flexible PEG block enhances their mobility and facilitates their crystallization, in agreement with our previous study on the same family of copolymers, in which the crystalline fraction of PLA increased from ~3% to 20–26% in the triblocks [27]. The moderate decrease in *T*_m_ with increasing MW_PEG_ suggests, at the same time, the formation of smaller and/or less perfect crystals.

The thermal properties of the polymeric matrices were closely associated with the melatonin release behaviour of the formulations. The incorporation of PEG into the PLLA–PEG–PLLA triblock copolymers progressively reduced the glass transition temperature from 53 °C for neat PLA to 46, 39, and 35 °C for PLLA–PEG–PLLA_4000, _6000, and _8000, respectively, indicating the enhanced mobility of the amorphous polymer phase and a plasticization effect. This effect, combined with the hydrophilicity of the PEG segments, is expected to promote water uptake, matrix plasticization, and the diffusion of melatonin during dissolution. Accordingly, the formulations containing the copolymers with longer PEG blocks, F1 and F2, showed the most rapid release, reaching approximately 90% release after 300–480 min, whereas the neat PLA-containing formulation F3 released only approximately 65% after 480 min. Although the copolymers exhibited higher crystallization enthalpies and slightly lower melting temperatures than neat PLA, the accelerated release suggests that PEG-mediated hydration and the reduced rigidity of the amorphous phase were more influential than crystallinity alone. The slower release observed for F5 and F6, despite the presence of PEG-containing copolymers, further indicates that the higher HPMC K15M content formed a stronger hydrated gel barrier, partially counteracting the enhanced water uptake and polymer chain mobility induced by PEG.

The hydrolysis rate under non-enzymatic and enzymatic conditions was heavily dictated by the molecular weight of the hydrophilic PEG block. The incorporation of PEG segments accelerated mass loss without the addition of enzymes, and the effect increased with PEG molecular weight, reaching 6.30 ± 0.28%, 8.70 ± 1.40% and 11.03 ± 0.83% for PLLA-PEG-PLLA_4000, _6000 and _8000, respectively. This behaviour is highly characteristic of what is theoretically expected from a bulk erosion mechanism, wherein water molecules are anticipated to penetrate the entire polymer matrix and initiate internal bond cleavage prior to complete mass loss. Because the larger PEG blocks impart exceptional hydrophilicity to the polymer matrix, water molecules easily penetrate the internal structure of PLLA-PEG-PLLA_8000. This widespread water penetration leads to the simultaneous hydrolytic cleavage of the ester bonds in the amorphous PLA segments throughout the entire bulk of the material [38]. Compared with hydrolysis alone, the presence of the enzymes had only a limited effect on pristine PLA but increased the mass loss of the PEG 4000–8000 copolymers about 2–2.5-fold. As the enzymatic degradation of PLA proceeds primarily by surface erosion alongside water penetration, its overall extent depends heavily on the wettability and the dynamically evolving accessible surface area of the film [39]. These combined factors significantly accelerate the degradation profile, leading to faster degradation kinetics in the presence of the enzyme. The hydrophilic, phase-separated surfaces of the higher-molecular-weight PEG segments promote localized swelling and micro-pitting. This effectively increases the molecular surface area accessible to the enzyme [38]. Such behaviour is particularly important under conditions resembling gastrointestinal exposure, where concurrent water uptake and enzymatic activity may progressively increase matrix porosity and contribute to the sustained release of melatonin from the oral dosage forms.

In the dissolution tests, formulations F1 and F4 released MLT in a similar manner, with over 80% of the hormone released after 240 min. Owing to this comparatively rapid release, both formulations may be regarded as promising candidates for the further development of dosage forms targeting sleep onset difficulties. Interestingly, although F1 and F4 differ in composition (PLLA-PEG-PLLA 6000 triblock in F1 and PLLA-PEG-PLLA 4000 blend in F4) [40], their MLT release profiles almost overlap. A possible explanation is that both the PLLA-PEG-PLLA 6000 triblock (F1) and the PLLA-PEG-PLLA 4000 blend (F4) form the same type of intermolecular bonds, mainly hydrogen bonds, with the C5-methoxy group and the C3-alkanamido side chain of melatonin; such interactions are known to improve the solubility of the hormone [10]. Nevertheless, the two formulations follow different release kinetics, in line with their different compositions: F1 exhibits an n value of 0.96, corresponding to a Super Case II transport mechanism, whereas F4 shows n = 0.84, indicative of anomalous (non-Fickian) transport.

Formulations F2 and F3 provided a more prolonged release of MLT, with approximately 84% of the hormone released after 480 min. Their release behaviour resembled that of Circadin^®^, the marketed prolonged-release melatonin product, suggesting that they could serve as a basis for formulations designed to sustain MLT release over longer periods. For F2, an n value of 0.23 was obtained; such low values (n < 0.45) are often reported for cylindrical tablets and are ascribed to pseudo-Fickian diffusion, typically arising from the structural heterogeneity of the matrix or from transport occurring in more than one dimension [41]. In contrast, F3 yielded n = 0.79, pointing to anomalous (non-Fickian) transport.

Formulations F5 and F6, which contain the PLLA-PEG-PLLA 8000 and PLLA-PEG-PLLA 4000 copolymers, respectively, gave nearly overlapping MLT release profiles, even though the two copolymers differ in solubility (moderate for the former and very high for the latter). On the other hand, water diffuses more readily into PLLA-PEG-PLLA 8000. The similar release of melatonin from these two formulations may therefore be related to the equal amounts of the other excipients they contain, implying that the two PLA-PEG copolymers exert a comparable degree of control over MLT release under the conditions studied. Both formulations follow anomalous (non-Fickian) transport (n = 0.75 for F5 and 0.70 for F6), meaning that two processes take place simultaneously, namely drug diffusion and the relaxation/swelling of the polymer chains.

Overall, the incorporation of longer PEG blocks increased the hydrophilicity of the PLLA–PEG–PLLA copolymers and exerted a plasticizing effect, as evidenced by the progressive decrease in their Tg. Upon exposure to the dissolution medium, these characteristics facilitated water penetration and the hydration-induced plasticization of the PLA-rich matrix, thereby promoting the formation of aqueous diffusion pathways for melatonin release. Accordingly, the copolymer-containing formulations exhibited substantially faster drug release than the neat PLA formulation; however, the release rate did not increase strictly monotonically with PEG molecular weight, indicating that additional formulation-related factors may also influence the overall release behaviour.

## 5. Conclusions

In the present study, PLLA–PEG–PLLA triblock copolymers bearing PEG blocks of 4000–8000 g/mol were prepared via ring-opening polymerization and assessed, for the first time, as excipients for modulating MLT release from matrix tablets. Although the PEG content was low, PEG block length markedly influenced the properties of PLA, decreasing the glass transition temperature, enhancing crystallization and speeding up both hydrolytic and enzymatic degradation. By using these copolymers, the MLT release profile could be tuned. F1 and F4 delivered the hormone quickly and could be appropriate for sleep onset problems, while F2, F3, F5 and F6 appear promising for formulations that target both sleep initiation and sleep maintenance. Nevertheless, the release rate was not a monotonic function of PEG molecular weight, which suggests that copolymer crystallinity and the amount of hydrophilic excipients, such as HPMC K15M, also govern the release behaviour.

Further studies should therefore seek to separate the plasticizing effect of the PEG block from its nucleating effect, e.g., by adjusting the LA/PEG ratio or by incorporating amorphous blocks based on D,L-lactide, thereby allowing the release rate to be predictably tuned through the copolymer composition. In addition, release experiments under changing pH and biorelevant conditions should be performed, together with the monitoring of how tablet swelling, matrix erosion and polymer crystallinity evolve during dissolution and on storage, in order to elucidate the release mechanism and determine the shelf-life of the formulations. Owing to their reduced *T*_g_, the copolymers are also promising for low-temperature processing, for instance by hot-melt extrusion or 3D printing, as well as for bilayer tablets in which an immediate- and a prolonged-release layer are combined in one dosage form. Lastly, in vivo pharmacokinetic investigations are needed to verify whether the in vitro release profiles result in plasma MLT concentrations that reproduce the natural nocturnal secretion pattern of the hormone.

## Figures and Tables

**Table 1:** Composition (% *w*/*w*) of MLT formulations.

Ingredients	F1	F2	F3	F4	F5	F6
Melatonin	1	1	1	1	1	1
Neat PLA			25			
PLLA-PEG-PLLA_4000				25		25
PLLA-PEG-PLLA_6000	25					
PLLA-PEG-PLLA_8000		25			25	
HPMC K15M	19	19	8	8	38.5	38.5
Sodium Alginate	38.5	38.5	45	45	16.5	16.5
Lactose Monohydrate	5.5	5.5	10	10	8	8
Microcrystalline Cellulose	10	10	10	10	10	10
Magnesium Stearate	1	1	1	1	1	1

**Table 2:** Molar LA/PEG ratios, molecular weight (g/mol) and polydispersity index of synthesized samples.

Sample	LA/PEG Molar Ratio	Mn (g/mol)	PDI
PLA neat	640/1	82 k	1.81
PLLA_PEG_PLLA_4000	640/1	108 k	1.85
PLLA_PEG_PLLA_6000	640/1	62 k	2.30
PLLA_PEG_PLLA_8000	640/1	101 k	2.32

**Table 3:** The thermal transitions of neat PLA and the PLLA-PEG-PLLA triblock copolymers, as determined by DSC (scan 2): glass transition (*T*_g_) and melting (*T*_m_) temperatures from the heating scan at 10 K/min; crystallization temperature (*T*_c_) and enthalpy (Δ*H*_c_) from the cooling scan at 20 K/min as well as the corresponding crystalline fraction (CF).

Sample	T_g_ (°C)	T_c_ (°C)	Δ_Hc_ (J/g)	T_m_ (°C)	CF (%)
Neat PLA	53	96	2	176	2.15
PLLA_PEG_PLLA_4000	46	93	8	174	8.6
PLLA_PEG_PLLA_6000	39	95	24	169	25.8
PLLA_PEG_PLLA_8000	35	91	30	166	32.2

**Table 4:** The kinetic parameters and correlation coefficients (R^2^) obtained by fitting the zero-order, first-order, Higuchi and Korsmeyer–Peppas models to the release data of the prepared formulations and Circadin^®^.

	Zero-Order		First-Order			Higuchi		Peppas		
Formulation	R^2^	K_0_	R^2^	Y_1_	K_1_	R^2^	K_H_	n	K_KP_	R^2^
F1	0.84	0.19	0.72	35.20	−0.002	0.89	4.63	0.96	0.46	0.96
F2	0.95	0.18	0.83	21.63	−0.003	0.88	3.53	0.23	0.23	0.99
F3	0.95	0.17	0.84	26.94	−0.003	0.93	3.88	0.79	0.83	0.98
F4	0.89	0.19	0.78	32.61	−0.003	0.92	4.49	0.84	0.76	0.99
F5	0.99	0.08	0.94	10.61	−0.003	0.92	1.77	0.75	0.43	0.99
F6	0.98	0.11	0.99	15.41	−0.003	0.90	2.48	0.70	0.82	0.95
Circadin^®^	0.97	0.11	0.90	23.20	−0.002	0.97	3.02	0.55	2.35	0.98

## Data Availability

The original contributions presented in this study are included in the article. Further inquiries can be directed to the corresponding author.

## Abbreviations

The following abbreviations are used in this manuscript:

MLT	melatonin
PLA	polylactic acid
PEG	polyethylene glycol
